# m^6^A RNA Methylation in Insect Biology: A Bibliometric Analysis with a Focus on METTL3

**DOI:** 10.3390/insects17070703

**Published:** 2026-07-07

**Authors:** Jiayang Zhang, Xinyue Huang, Xiaolei Wu, Yihan Lin, Wenmei Wu

**Affiliations:** 1 School of Life Sciences and Biopharmaceutics, Guangdong Pharmaceutical University, Guangzhou 510006, China; 2112443047@stu.gdpu.edu.cn (J.Z.); 2300930213@stu.gdpu.edu.cn (X.H.); 2112543059@stu.gdpu.edu.cn (X.W.); 2300930123@stu.gdpu.edu.cn (Y.L.); 2 Guangdong Provincial Key Laboratory of Advanced Drug Delivery, Guangdong Provincial Engineering Center of Topical Precise Drug Delivery System, Guangdong Pharmaceutical University, Guangzhou 510006, China

**Keywords:** METTL3, m^6^A, methylation modification, insect developmental regulation, bibliometrics

## Abstract

RNA *N*^6^-methyladenosine (m^6^A) modification is a pivotal post-transcriptional regulator of diverse biological processes. Despite the growing interest in insect epitranscriptomics, a systematic evaluation of research trends and hotspots remains lacking. Here, we conducted a bibliometric analysis to map the global landscape of METTL3 and m^6^A research in entomology over the past decade. Our results reveal a steady increase in publications, signaling a phase of rapid expansion in this field. Notably, the number of studies on METTL3 is significantly lower than that of general m^6^A research, suggesting that current efforts prioritize phenotypic over the mechanistic roles of core regulatory components. Keyword co-occurrence analysis identifies *Bombyx mori*, *Locusta migratoria*, and *Drosophila melanogaster* as the primary model systems. Research hotspots predominantly center on METTL3-mediated regulation of development, behavioral plasticity, immunity, and host–pathogen interactions. These findings highlight insect METTL3 as a burgeoning research frontier. Future studies should emphasize cross-species comparisons and the systematic dissection of regulatory networks to provide novel theoretical frameworks and molecular targets for sustainable pest management and resource insect utilization.

## Simple Summary

RNA methylation is a key mechanism controlling gene expression. This study used bibliometric analysis to examine research on this process, particularly the METTL3 protein, in insects over the past decade. The results show increasing publications, but METTL3-focused studies remain far fewer than general RNA methylation research. Key model insects include *Bombyx mori*, *Locusta migratoria*, and *Drosophila melanogaster*. Research hotspots center on METTL3’s roles in development, behavior, immunity, and host–pathogen interactions. These findings highlight METTL3 as an emerging frontier in insect science. Future cross-species comparisons and network-level studies may support sustainable pest management and better use of beneficial insects.

## 1. Introduction

Epitranscriptomics, a frontier discipline in the life sciences, profoundly elucidates the critical role of RNA chemical modifications in the regulation of gene expression [1]. Among the diverse array of known modifications, *N6*-methyladenosine (m^6^A) stands out as the most prevalent internal epigenetic mark on eukaryotic messenger RNA, demonstrating immense research potential within the Class Insecta—the most taxonomically diverse group of animals [2,3]. As the primary catalytic subunit of the m^6^A methyltransferase complex (“writer”), METTL3-mediated modification functions as much more than a basal metabolic event. Recent evidence underscores its role as a sophisticated regulatory hub, orchestrating complex physiological activities and evolutionary adaptations in insects, ranging from developmental plasticity to environmental stress responses [4,5].

In recent years, research on insect METTL3 has rapidly progressed from basic gene function identification to in-depth analysis of complex biological phenotypes. Accumulating evidence indicates that METTL3 as the core of the m^6^A methyltransferase complex, exerts multifaceted regulatory effects across diverse insect taxa [2,4,6]. Specifically, METTL3 serves as a key modulator of behavioral plasticity; in the migratory locust (*Locusta migratoria*), its expression fluctuates in response to population density, thereby orchestrating aggregation and locomotor behaviors. Beyond behavior, METTL3 is indispensable for growth and metamorphosis [6]. For example, in the silkworm (*Bombyx mori*), silencing the “writer” components METTL3 or METTL14 delays molting by disrupting ecdysone signaling [7]. Furthermore, METTL3 mediates host–plant adaptation and antiviral immunity; in *Plutella xylostella*, its deficiency impairs larval fitness during host shifts, while in silkworms, METTL3 is upregulated upon nucleopolyhedrovirus (NPV) infection to suppress viral replication [4]. Notably, METTL3 has emerged as a critical factor in insecticide resistance. It has been shown to modify transcripts of cytochrome P450 genes, thereby conferring neonicotinoid resistance in whiteflies [8]. Collectively, these breakthroughs position METTL3 as a functional bridge linking epigenetic modification to environmental fitness in insects.

As research evidence continues to accumulate, the field of insect METTL3 is entering a phase of rapid expansion. In this context, traditional qualitative reviews face challenges in objectively and quantitatively delineating the global landscape, the intricate evolution of knowledge structures, and the cross-disciplinary dynamics of the field. To address this gap, bibliometrics, a quantitative analytical framework grounded in mathematics and statistics, can objectively uncover the distribution of research capabilities, collaboration networks, the evolution of hot topics, and future development trends within a discipline by mining the external characteristics of vast amounts of literature data [9].

Therefore, this study represents the first attempt to systematically organize and visually analyze the insect METTL3 research domain using bibliometric methodologies. By synthesizing a decade of global data, this analysis provides a macro-perspective and data-driven insights into insect physiology, integrated pest management and innate immunology. Furthermore, the findings offer a robust theoretical foundation for the development of next-generation pest control strategies centered on epitranscriptomic intervention, thereby bridging the gap between basic epigenetic research and sustainable agricultural applications.

## 2. Methods

### 2.1. Objectives

**Figure 1:**
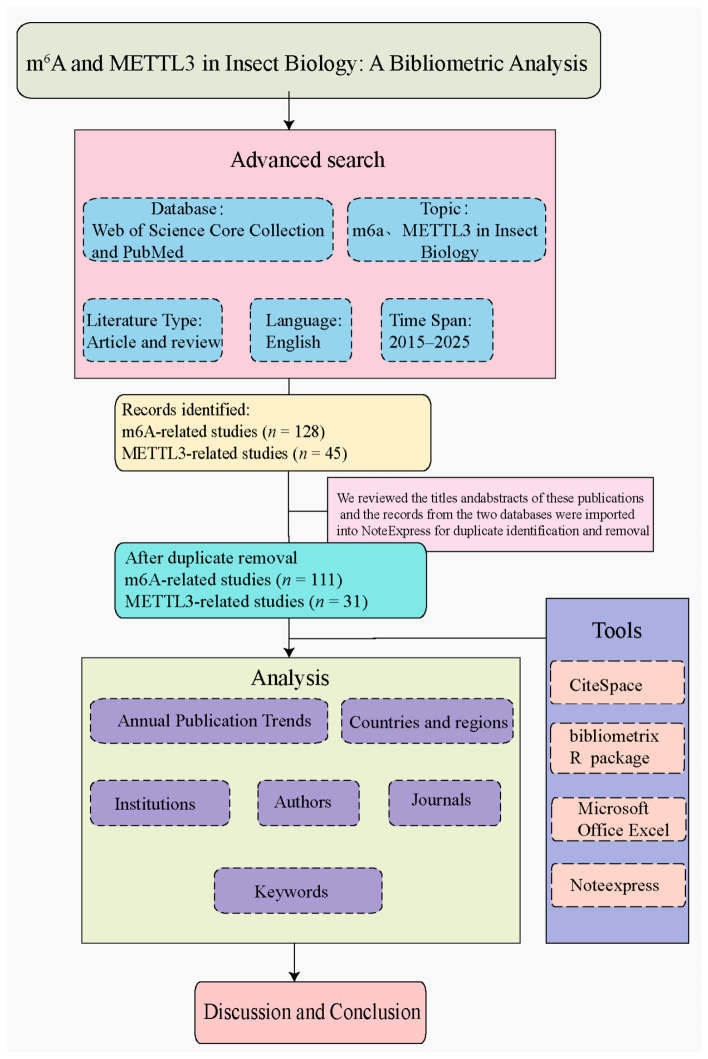
PRISMA flow diagram showing the selection of studies related to METTL3 and m^6^A in insects.

The primary objective of this study was to construct a multidimensional knowledge map of m^6^A and METTL3 research, specifically emphasizing their emerging regulatory role in insect biology. This was achieved through the analysis of co-cited references and keyword co-occurrence patterns. Secondary objectives were to quantify the collaborative research network (including countries, institutions, and authors), identify established and emerging research trends, and pinpoint potential gaps specifically within the insect research domain. A systematic flowchart of the bibliometric workflow is illustrated in Figure 1.

### 2.2. Search Strategy and Data Collection

Literature retrieval was performed using the Web of Science Core Collection and PubMed databases. To ensure data quality, the Web of Science index was restricted to the Science Citation Index Expanded. The search conditions were set as [TS = (METTL3) AND TS = (insect)] and [TS = (m^6^A methylation) AND TS = (insect)], with the time range defined from 1 January 2015 to 30 December 2025, with the language limited to English. Through screening, 128 and 45 records were obtained, respectively. These articles were saved in plain text format as “download.txt” and imported into CiteSpace software (6.4.R1), where duplicates were removed based on titles, authors, and DOIs; a final dataset of 111 and 31 unique articles was consolidated for subsequent analysis. The m^6^A dataset encompassed studies on writers (including METTL3, METTL14, WTAP), erasers (FTO, ALKBH5), and readers (YTHDF proteins), whereas the METTL3 dataset was restricted to studies specifically targeting METTL3.

### 2.3. Data Analysis

Publication trends were initially visualized using Microsoft Excel to assess the temporal distribution of literature output. Subsequently, an advanced bibliometric analysis was conducted using CiteSpace to uncover the intellectual structure of the field. The analytical parameters were configured as follows: the time slicing from January 2015 to October 2025, with a 1-year interval per slice. The threshold (top N per slice) was set to 50, meaning the 50 most frequently occurring nodes were selected in each time slice to ensure the continuity and representativeness of the network structure. To minimize noise, frequency filtering was applied (keyword co-occurrence frequency ≥ 3, reference co-citation frequency ≥ 2). Meaningless terms (e.g., generic numbers or stop words) were removed, and synonyms (e.g., “*N*^6^-methyladenosine” and “m^6^A”) were merged to ensure data integrity. The network size K-value (G-index) was set to 25, and the Pathfinder algorithm was used for network pruning to enhance clarity. The Log-Likelihood Ratio algorithm was utilized for keyword clustering. Visualizations generated included author/institutional collaboration networks, keyword clustering maps, and timezone views, providing a comprehensive data-driven interpretation of the insect epitranscriptomic landscape (Figure 1).

## 3. Results

### 3.1. Publication Trend Analysis

**Figure 2:**
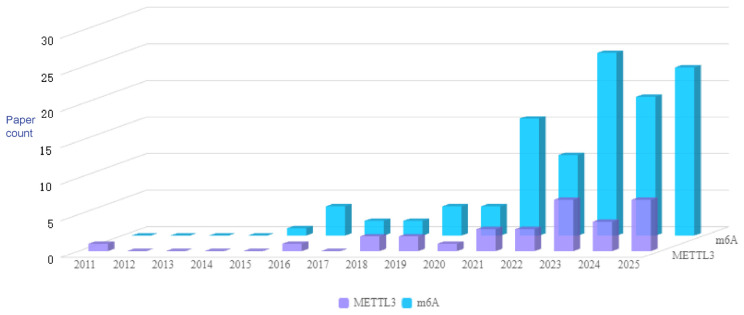
Annual publication volumes of METTL3-related literature compared with m^6^A studies.

The number of publications related to METTL3 and m^6^A in insect research has shown an overall increase over time, although clear differences are observed in their growth patterns and research intensity. From 2011 and 2016, only a limited number of studies were published, indicating that investigations into METTL3 and m^6^A modifications in insects were still at an early stage exploratory stage. Since 2017, m^6^A-related publications have increased markedly, with a particularly pronounced rise after 2021, suggesting that m^6^A, as an important RNA epigenetic modification, has gradually emerged as a major research hotspot in insect studies. In contrast, although publications on METTL3 have also shown a steady increase over time, their overall number remains substantially lower than that of m^6^A, and the growth rate is relatively moderate (Figure 2). These trends indicate that current insect research is predominantly focused on the global functions of m^6^A modification, whereas studies specifically targeting its core “writer” enzyme, METTL3, remain limited.

### 3.2. Author Collaboration Analysis

In this network, nodes symbolize authors, with their magnitude proportional to publication output. Edges denote collaborative ties, where greater edge density reflects more intensive cooperation. Node color encodes the temporal dimension, transitioning from cool to warm tones to trace the progression from early to recent studies. Unlabeled nodes still represent authors within the collaboration network, but their labels were omitted automatically due to lower publication frequency or dense network distribution in order to maintain visual clarity.

**Figure 3:**
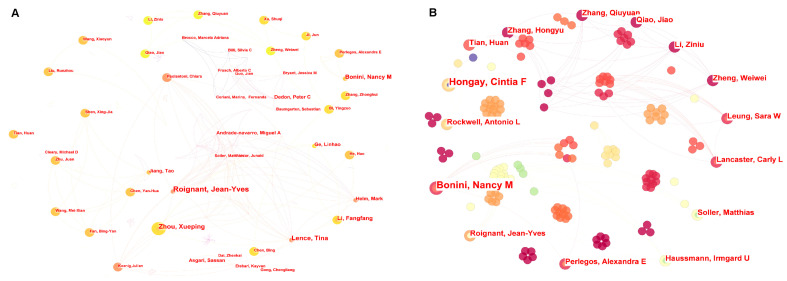
Co-authorship network of research on m^6^A and METTL3 in insects. (**A**) Co-authorship network of research on m^6^A in insects. (**B**) Co-authorship network of research on METTL3 in insects.

Viewed holistically, both m^6^A- and METTL3-related studies have coalesced into several relatively concentrated collaboration clusters. However, the global network connectivity remains limited, exhibiting a pattern characterized by multiple cores with weak interconnections. In m^6^A research, relatively stable collaboration networks have emerged, represented by authors such as Jean-Yves Roignant and Nancy M. Bonini, whose research encompasses RNA methylation regulation, development, and behavior (Figure 3A). In contrast, the author network of METTL3 studies in insects is smaller in scale and more fragmented, primarily anchored by research groups led by Qiuyuan Zhang, Ziniu Li, and Jiao Qiao, with a focus on METTL3-associated molecular mechanisms and its roles in insect development (Figure 3B). Overall, m^6^A research in insects boasts a more established and mature collaboration structure. METTL3 studies are still in an emergent phase and have yet to form robust international networks, suggesting considerable space for further expansion.

### 3.3. Institutional Collaboration Analysis

**Figure 4:**
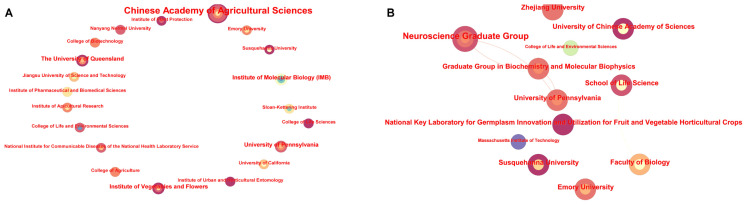
Institutional collaboration analysis. (**A**) Collaboration network of institutions in insect m^6^A research. (**B**) Collaboration network of institutions in insect METTL3 research.

Institutions involved in insect m^6^A research are primarily concentrated within universities, agricultural academies, and key laboratories focusing on biology, life sciences, and entomology (Figure 4A). The collaboration network demonstrates a moderate level of connectivity, with several influential institutions occupying central positions, particularly the Chinese Academy of Agricultural Sciences and major university-affiliated life science departments. These institutions exhibit relatively high node prominence, reflecting their substantial research contributions and academic influence in the field. Nevertheless, inter-institutional cooperation remains regionally clustered, and large-scale international collaboration networks have yet to be fully established.

In Figure 4B, the METTL3-related institutional network is comparatively smaller but displays stronger local cohesion around several core institutions, including Zhejiang University and university-affiliated medical and biological research centers. Compared with the broader m^6^A institutional network, METTL3 research demonstrates a more concentrated collaboration pattern, with interactions primarily centered on molecular biology, biochemistry, and functional regulatory studies. This difference suggests that insect m^6^A research is currently characterized by broad exploratory development, whereas METTL3-related studies have gradually evolved toward more specialized and mechanistically focused research directions.

### 3.4. Country-Level Collaboration Analysis

**Figure 5:**
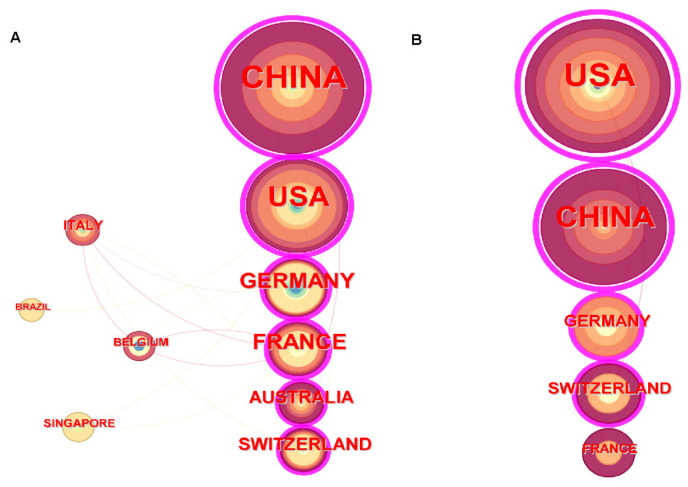
Country-level collaboration analysis. (**A**) Country co-occurrence network of research on m^6^A in insects. (**B**) Country co-occurrence network of research on METTL3 in insects.

Countries including Germany, France, Australia, and Switzerland have established cohesive research consortia and sustain strategic collaborative ties with China and the United States. The collaboration network is centered on China and the United States, which exhibit the highest publication output and the greatest number of international collaborative links., yet complemented by extensive involvement from Europe and other regions (Figure 5A).

In contrast, the geographical distribution of METTL3-related insect research is markedly more condensed, with China and the United States retaining their status as the predominant contributors (Figure 5B). Notably, the United States displays a marginally greater node magnitude, underscoring its enduring leadership in this specialized domain. Compared to the broader m^6^A landscape, the METTL3 country-level network is more constrained in scale and involves fewer constituent nations; however, the stronger connections among the core countries indicate a relatively concentrated pattern of international collaboration within the METTL3 research community.

### 3.5. Keyword Co-Occurrence Analysis

Keywords effectively reflect the thematic focus of the literature and the developmental trends of research, offering an incisive synthesis of the primary topics within a specific domain. By leveraging keyword co-occurrence networks analysis, CiteSpace facilitates a systematic mapping of the current research landscape. This approach elucidates the major themes and burgeoning trends associated with RNA methyltransferase METTL3, thereby furnishing a strategic roadmap for future investigations.

#### 3.5.1. Co-Occurrence Network Analysis

**Figure 6:**
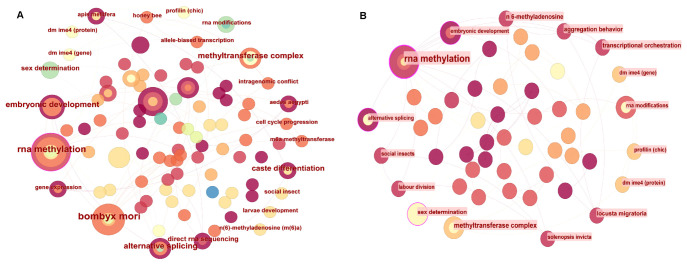
Keyword co-occurrence analysis. (**A**) Keyword co-occurrence network of research on m^6^A in insects; (**B**) keyword co-occurrence network of research on METTL3 in insects.

The keyword co-occurrence reveals the knowledge structure of m^6^A-related research in insects. Each node represents a keyword, and node size reflects its occurrence frequency. Links between nodes indicate co-occurrence relationships. Different colors represent distinct thematic clusters. Unlabeled nodes correspond to lower-frequency keywords or nodes whose labels were automatically hidden by the visualization software to improve readability. “RNA methylation” and “*N*^6^-methyladenosine”, occupy central positions in the network, characterized by large node sizes and dense connections, indicating that RNA methylation represents the core theme of this field. Meanwhile, keywords related to development and behavior, including “embryonic development”, “sex determination”, and “aggregation behavior”, are distributed around the core nodes, reflecting the major biological contexts in which m^6^A has been investigated (Figure 6A). In the METTL3-focused keyword co-occurrence network, “RNA methylation” remains the most prominent core keyword and shows strong co-occurrence with terms such as “*N*^6^-methyladenosine”, “RNA modification”, and “methyltransferase complex”, underscoring the central role of METTL3 as the catalytic component of the m^6^A methyltransferase machinery. Compared with the global m^6^A network, the METTL3 network shows a higher concentration of keywords related to specific biological processes and insect models, including “embryonic development”, “labour division”, and “sex determination”, as well as species including *Locusta migratoria* and *Solenopsis invicta* (Figure 6B).

Collectively, these networks delineate the overall conceptual framework of m^6^A research in insects and its more specialized extension at the METTL3 level. While studies on m^6^A emphasize a broad landscape of RNA modification mechanisms, METTL3-centered research is more tightly linked to functional analyses of insect development, behavior, and species-specific characteristics. Notably, compared with the broader m^6^A network, the METTL3-focused network contains fewer generic regulatory terms and a higher proportion of phenotype- and species-oriented keywords, suggesting a gradual shift from predominantly mechanistic exploration toward functional and organism-level investigations.

#### 3.5.2. Keyword Clustering Analysis

**Figure 7:**
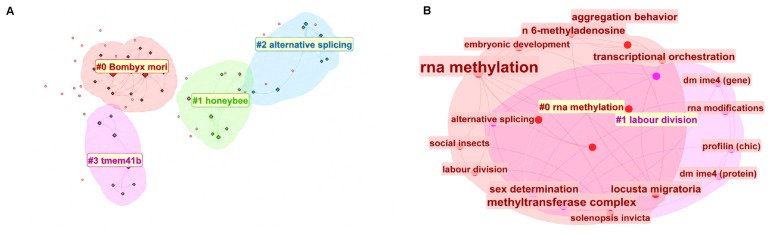
Keyword clustering analysis. (**A**) Keyword clustering analysis of research on m^6^A in insects; (**B**) keyword clustering analysis of research on METTL3 in insects.

The topological characteristics of the keyword clustering network (Figure 7A) reveal well-defined modularity, signifying that the insect m^6^A research field has matured into several stable thematic domains, cluster #0 *Bombyx mori* emerges as the largest and most cohesive grouping, underscoring the central role of the silkworm as a preeminent model insect in m^6^A modification research. The dense internal connections within this cluster suggest that studies based on *B. mori* are thematically focused and have progressively expanded in-depth functional characterization. In addition, cluster #1, “honeybee”, and cluster #2 “alternative splicing”, identify two critical research trajectories, with the former primarily interrogates the epigenetic regulation of social behavior and caste development, and the latter emphasizes the mechanistic nexus between m^6^A and post-transcriptional processing. Notably, Cluster #3 “TMEM41B”, although smaller in scale, exhibits high thematic specificity, potentially representing an emerging frontier focused on specific molecular transporters or host–virus interaction factors.

The thematic interconnection map (Figure 7B) of Keyword clustering further elucidates the hierarchical structure of the field. “RNA methylation” occupies a central position in the network and is strongly associated with keywords such as “m^6^A (*N*^6^-methyladenosine)”, “RNA modifications”, and “methyltransferase complex”, collectively constituting the theoretical core. Radiating from this foundation, research has progressively expanded toward functional dimensions, encompassing biological processes such as alternative splicing, embryonic development, sex determination, and social insect biology. Concurrently, the close proximity of species-related keywords, including *Locusta migratoria* and *Solenopsis invicta*, are closely linked to functional themes, indicating that current research is extending from molecular modification mechanisms toward specific insect species and their phenotypic regulation, reflecting a transition from fundamental biochemical mechanisms and applied ecological phenotypes.

#### 3.5.3. Timeline Analysis

**Figure 8:**
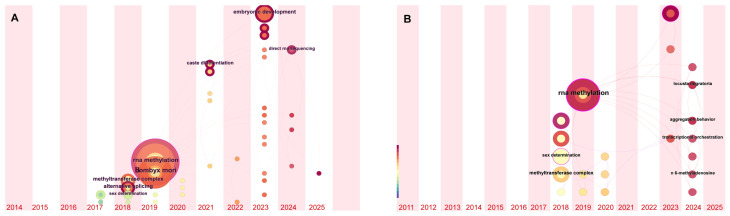
Timeline analysis. (**A**) Timezone view of keyword co-occurrence for m^6^A research in insects; (**B**) Timezone view of keyword co-occurrence for METTL3 research in insects.

Timeline analysis provides a longitudinal perspective on the shifting paradigms within insect m^6^A research. During the nascent phase (approximately 2017–2019), the field was characterized by a focus on fundamental biochemical frameworks, including the characterization of methyltransferase complexes and their foundational roles in sex determination (Figure 8A). These early efforts were predominantly anchored in the classical model insect *Bombyx mori*, reflecting an era of exploring the basic molecular composition of m^6^A machinery. Subsequently, a significant thematic expansion occurred after 2021; research hotspots diversified into higher-order biological processes, such as embryonic development, caste differentiation, and behavioral regulation, accompanied by the introduction of advanced methodologies such as direct RNA sequencing. These changes indicate that insect m^6^A research has expanded from mechanistic exploration toward complex phenotypic outcomes and high-resolution regulatory processes, showing a progressive trajectory from molecular modification to developmental processes and ultimately to behavior and phenotype.

The temporal trajectory of METTL3-specific research exhibits a discernible lag compared to general m^6^A trends, yet follows a similar path toward functional depth (Figure 8B). In more recent years (after 2022), research attention has increasingly converged on themes such as *N*^6^-methyladenosine, transcriptional orchestration, and aggregation behavior, while gradually extending to non-model insects including *Locusta migratoria*. This evolution suggests that METTL3 research has matured from enzymatic characterization to its current standing as a central regulator of behavioral plasticity and transcriptional network remodeling, reflecting a deepening research pathway from enzymatic characterization to biological function.

## 4. Molecular Foundations for Understanding m^6^A Dynamics in Insects

Within the intricate epitranscriptomic regulatory network of eukaryotes, RNA modifications have emerged as a cornerstone of transcriptomics research. To date, over 170 distinct types of RNA modifications have been cataloged. Among these, methylation modifications—notably by *N*^6^-methyladenosine (m^6^A), alongside 5-methylcytosine (m^5^C) and N^1^-methyladenosine (m^1^A)—play a decisive role in governing RNA metabolism. These modifications collectively form a multi-dimensional and sophisticated network that ensures the precise spatiotemporal regulation of gene expression [10]. These modifications are covalently and site-specifically incorporated into RNA molecules, thereby orchestrating a series array of post-transcriptional processes, including alternative splicing, mRNA stability, and translational efficiency. Among them, m^6^A emerges as the most prevalent and widely distributed internal modification in eukaryotic mRNA, representing a functional cornerstone of the epitranscriptomic landscape. The dynamic interplay between its enzymatic deposition (“writing”) and removal (“erasing”) constitutes a fundamental layer of gene expression control, providing a rapid and reversible mechanism for cellular adaptation [11].

### 4.1. m^6^A Methylation and Its Molecular Mechanism

The establishment, removal, and functional execution of m^6^A modification constitute a complex yet orderly dynamic reversible process, primarily reliant on the orchestrated interplay of three functional protein categories: “Writers”, “Erasers”, and “Readers”. The “Writer” constitute the core methyltransferase complex, which identifies conserved motifs within target RNA substrates and utilizes *S*-adenosylmethionine (SAM) as a methyl donor to catalyze the covalent linkage of a methyl group to the m^6^A position of adenine. The “Eraser”, comprising m^6^A demethylases, mediates the precise removal of these marks through precise demethylation reactions, thereby ensuring the reversibility and plasticity of the epitranscriptomic landscape. “Readers” are specialized proteins harboring conserved recognition modules, such as the YTH domain, that specifically bind to m^6^A sites [12], By recruiting downstream effector complexes, these proteins translate the m^6^A code into specific biological outcomes, such as altered RNA stability, splicing, or translation.

Collectively, “Writers”, “Erasers”, and “Readers” form a complete and conserved regulatory circuit of “modification establishment–dynamic removal–functional transmission” [13]. This integrated system empowers m^6^A-mediated gene regulation with remarkable plasticity, allowing for rapid spatiotemporal responses to both intrinsic physiological cues and extrinsic environmental signals. By facilitating the precise modulation of downstream transcriptomes, this circuit underpins an array of fundamental biological processes, ranging from cellular differentiation and organismal development to innate immune homeostasis and the pathogenesis of complex diseases.

### 4.2. Specific Regulation of m^6^A Methylation

The deposition of m^6^A methylation is orchestrated by a multi-subunit methyltransferase complex, termed the “Writer” machinery. The catalytic core of this complex is an obligate heterodimer composed of METTL3 and METTL14. In this architecture, METTL3 functions as the sole catalytic engine, harboring the active site for methyl transfer. In contrast, METTL14, despite lacking intrinsic enzymatic activity, serves as an indispensable scaffold. It facilitates substrate recognition by binding specific RNA motifs and stabilizes the overall complex conformation to enhance catalytic efficiency. This synergistic assembly ensures the highly precise and site-specific deposition of m^6^A marks across the transcriptome [14].

This precise structural specialization enables the METTL3-METTL14 heterodimer to exhibit a distinct sequence preference for RNA substrates, forming the molecular basis for the targeted regulation conferred by m^6^A and contributing to its high degree of specificity [15]. The heterodimer specifically recognizes and binds to the conserved RRACH motif (R = A/G and H = A/C/U), which serves as the primary docking site for initiating methylation. This sequence-guided recruitment machinery to discriminate specific RNA transcripts within the vast transcriptome, thereby providing a functional scaffold for the subsequent recruitment of “Reader” proteins and the ensuing metabolic cascades. Ultimately, the robust regulatory axis formed by the METTL3-METTL14 interaction guarantees both the fidelity and catalytic throughput of the modification process [16].

As the predominant internal RNA modification in the eukaryotic epitranscriptome, m^6^A functions as a highly dynamic and reversible regulatory axis rather than a static chemical mark [17,18]. This inherent plasticity allows m^6^A to act as a sensitive molecular switch, orchestrating real-time responses to a broad spectrum of intrinsic and extrinsic signals, including cell cycle progression, developmental transitions, and environmental perturbations such as nutrient deprivation or oxidative stress. By modulating the entire lifecycle of RNA transcripts—encompassing processing, transport, stability, and translational competency—m^6^A facilitates precise spatiotemporal control over gene expression. Consequently, this modification is indispensable for maintaining metabolic homeostasis, driving organismal development, and conferring the resilience required for adaptive responses to adverse environments [19].

### 4.3. Structure and Function of the Methyltransferase METTL3

As the cornerstone of the “Writer” complex in the m^6^A modification process, the methyltransferase acts as an essential molecular machine responsible for initiating m^6^A formation [20]. METTL3 serves as the catalytic nucleus, directly orchestrating targeting specificity, enzymatic efficiency, and the overall initiation of the m^6^A dynamic regulatory system. The functional execution of METTL3 is strictly dependent on the formation of a stable heterodimer with METTL14 [21]. In this architectural assembly, METTL3 acts as the primary catalytic driver, providing the enzymatic activity required for methyl transfer, while METTL14 functions as an essential structural scaffold that maintains the conformational stability of the complex [22].Their synergistic interaction is prerequisite for the precise recognition, sequence-specific binding, and efficient catalysis of target RNA substrates [14].

Structurally, METTL3 contains a conserved C-terminal methyltransferase domain, MT-A70, which serves as the binding site for the SAM molecule and is critical for catalyzing the methyl transfer reaction [23]. In-depth analysis of METTL3’s structural features using Uniprot 2025-7 and AlphaFold 3 software, reveals that the MT-A70 domain forms a “pocket-like” active center. Within the MT-A70 structure, multiple key amino acid residues perform distinct, well-coordinated roles to ensure the precision and efficiency of the methylation reaction [24]. Specifically, the Tyr395 residue is responsible for specifically recognizing the adenosine within the RRACH motif; Arg298 acts as an “anchoring point” for SAM binding, forming direct hydrogen bonds with the amino group of SAM; Asp343, mediated by a water molecule, interacts with the phosphate group of SAM to maintain its correct orientation within the active site; Gly300 and Ala302 form a hydrophobic pocket that encases the methyl donor portion of SAM, providing the necessary spatial environment for methyl transfer; Lys398 and Arg400 bind to the phosphate backbone of the mRNA and fix its conformation, effectively preventing motif mismatch. The C-terminal arginine-glycine-rich (RGG) repeat sequence of the auxiliary catalytic subunit METTL14 constitutes a secondary RNA-binding site This structural element not only significantly enhances the binding affinity of the heterodimer for RNA substrates but also contributes to stabilizing the catalytic domain of METTL3, being essential for maintaining the overall catalytic activity of the complex [25]. Together, this evolutionarily conserved enzymatic machinery forms the mechanistic foundation upon which the multifaceted regulatory roles of METTL3 in insect reproduction, behavior, immunity, and stress adaptation.

Furthermore, to achieve more precise spatiotemporal regulation of m^6^A modification, the METTL3-METTL14 heterodimer can interact with auxiliary proteins such as Wilms‘ tumor 1-associated protein (WTAP), vir like m^6^A methyltransferase associated (VIRMA), and zinc finger CCCH domain-containing protein 13 (ZC3H13) to assemble into a functional m^6^A methyltransferase holoenzyme complex, involved in the targeting and catalysis of methylation [26]. Within this complex, the interaction interface between WTAP and VIRMA forms a rigid “saddle-shaped” scaffold that restricts the binding of METTL3-METTL14 to non-specific DNA, thereby ensuring its catalytic activity is dedicated to RNA methylation [27].

Recent studies have revealed that METTL3 can also function independently of its m^6^A catalytic activity in certain contexts, for instance, by directly binding mRNA and participating in translational regulation. METTL3-mediated m^6^A modification participates extensively in a series of biological processes, including RNA splicing, nuclear export, stability maintenance, and translational efficiency regulation [28]. Consequently, it plays an indispensable regulatory role in various physiological and pathological processes, such as cell differentiation, embryonic development, metabolism, and tumorigenesis [29].

## 5. Research Progress on METTL3 in Insects

As the catalytic core component of the m^6^A methyltransferase complex, METTL3 serves as a pivotal regulator of diverse biological processes in insects, including growth, reproductive development, behavioral plasticity, and environmental adaptation [6]. Its mechanism of action involves mediating *N*^6^-methyladenosine (m^6^A) modification on RNAs, thereby influencing the methylation levels of mRNAs within metabolic pathways in insects. This subsequently regulates mRNA stability, splicing, and translational efficiency, ultimately affecting downstream signaling pathways and protein expression to achieve spatiotemporal fine-tuning of gene expression [5], ultimately driving complex phenotypic variations.

Emerging evidence highlights that METTL3 operates not merely as a regulator of isolated genes, but as a central node within a multi-tiered and interconnected network spanning from molecular modifications to organismal phenotypes. This regulatory architecture enables the precisely timed coordination of insect development, reproduction, and metabolic homeostasis. Furthermore, it dynamically modulates immune surveillance and stress resilience in a stage-specific manner, enhancing the organism’s fitness within fluctuating environments. By establishing a sophisticated physiological framework throughout the entire insect life cycle, METTL3 provides a fertile theoretical ground for advancing insect biology and developing novel biopesticide strategies.

### 5.1. Promotion of Insect Reproduction, Growth, and Development

Recent years have witnessed substantial breakthroughs in deciphering the molecular mechanisms by which METTL3-mediated m^6^A modification orchestrates insect reproductive development. Integrated evidence underscores that METTL3 expression is indispensable for male virility, female oogenesis, and successful embryogenesis across diverse insect taxa.

The pathways through which METTL3 governs insect fertility are multifaceted, ranging from hormonal modulation to structural remodeling. In the oriental fruit fly, *Bactrocera dorsalis*, BdMETTL3 exhibits high transcriptional abundance in the male accessory glands. Knockout of *BdMETTL3* leads to decreased titers of 20-hydroxyecdysone (20E, the active form of the molting hormone), resulting in testicular malformations and impaired sperm motility. Mechanistically, *BdMETTL3* catalyzes m^6^A deposition at five conserved RRACH motifs within the coding sequence (CDS) of Disembodied—a rate-limiting enzyme in the ecdysteroidogenic pathway—thereby enhancing its expression to maintain male reproductive vigor [30]. In *Drosophila*, METTL3 is essential for maintaining the architectural integrity of the germline. METTL3-deficient males exhibit meiotic perturbations and disorganized spermatid bundles, leading to terminal defects in spermiogenesis. This phenotype is largely attributed to the aberrant upregulation of Hsp608. During the transition from round spermatids to elongated, individualized sperm, the assembly of the actin-based individualization complex is paramount; METTL3 deficiency disrupts this assembly via Hsp608 interference, thereby aborting sperm individualization [31]. Furthermore, DmIme4, the homolog of METTL3 in *Drosophila*, recognizes and binds to specific site clusters within the precursor mRNA of the filamentous actin protein Chic in the testes. This interaction facilitates the folding of Chic transcripts into stable hairpin secondary structures, which not only promotes their alternative splicing but also augments protein synthesis. This regulatory axis is vital for maintaining the structural integrity of cyst cells, which form the protective niche required for germ cell differentiation [32].

Mirroring its critical function in males, METTL3 is equally indispensable for female reproductive fitness. In studies on *Tribolium castaneum*, females with disrupted METTL3 expression, when mated with normal males, failed to produce offspring and exhibited underdeveloped ovaries alongside significantly reduced egg quantity and quality [2]. Similarly, METTL3 knockdown in female *Drosophila* resulted in irrecoverable ovarian underdevelopment and egg-laying arrest. A primary mechanism involves ovary-enriched METTL3, which prolongs the half-life of Insulin receptor (*InR*) mRNA and promotes its translation. The resulting elevation in InR signaling triggers a regulatory cascade that activates ecdysone biosynthetic enzymes and their downstream responsive genes. This InR-20E cross-talk, orchestrated by METTL3, is fundamental for sustaining vitellogenesis and ensuring proper oocyte maturation [33].

Beyond its pivotal roles in gametogenesis and gonadal development, METTL3-mediated m^6^A modification serves as a cornerstone for insect embryogenesis by orchestrating critical signaling cascades. Transcriptomic analysis of eggs from *METTL3*-knockdown females revealed 112 downregulated genes significantly enriched in biological processes such as cellular development, transmembrane transport of organic acids, anatomical structure morphogenesis, and DNA-binding transcription factor activity [2]. This finding underscores that m^6^A modification is an indispensable requirement for embryonic viability and architectural patterning. While METTL3 functions as a key regulatory hub in this context, the precise molecular trajectories and specific downstream effectors it governs during early development represent a burgeoning frontier that remains to be fully charted.

Subsequent investigations have yielded mechanistic insights into how METTL3 coordinates these developmental programs. In adult female *Drosophila melanogaster*, the *METTL3* homolog, *DmIme4*, exhibits potent and tissue-specific expression within the ovaries. During oogenesis, DmIme4 is essential for the activation of the Notch signaling pathway, which drives the rapid proliferation of follicle cells—a prerequisite for proper egg chamber maturation [34]. Furthermore, in the silkworm *Bombyx mori*, transcriptomic profiling of METTL3-knockdown embryos revealed a massive reprogramming of the developmental landscape. The identified differentially expressed genes (DEGs) were significantly enriched in evolutionarily conserved pathways, including purine metabolism, the Toll/Imd immune axis, and the Wnt signaling cascade. These pathways represent the biochemical and inductive framework required for embryonic patterning and metabolic priming in silkworms [35].

Furthermore, METTL3-mediated m^6^A methylation displays a highly dynamic and stage-specific expression profile throughout the *Drosophila* life cycle. For instance, in female *Drosophila*, METTL3 abundance progressively accumulates during embryogenesis, undergoes a sharp decline during the larval and pupal stages, and ultimately peaks in the adult stage [33]. In *Apis mellifera*, m^6^A methylation levels were significantly downregulated in larvae, while a marked upregulation of m^6^A modification was detected in the brains of adult [36]. This temporal oscillation likely reflects the fluctuating demand for m^6^A-dependent modulation of biosynthetic pathways tailored to specific growth phases. In holometabolous insects, the metamorphosis from embryo to larva, pupa, and adult is characterized by the radical reprogramming of the transcriptomic landscape [37]. This dramatic transition provides a fundamental framework for investigating how METTL3 coordinates diverse physiological outputs through epigenetic fine-tuning.

The function of METTL3 transcends a singular molecular event; it serves as a central regulatory hub integrating epitranscriptomic signals, cellular fate decisions, and systemic physiological responses. Continued elucidation of METTL3 and its intricate regulatory networks not only establishes a new paradigm for understanding insect reproductive biology but also paves the way for innovative “epigenetic-based” pest management strategies. By targeting the molecular Achilles’ heel of insect development, such approaches offer a promising frontier for sustainable agricultural biotechnology.

### 5.2. Regulation of Biosynthesis and Metabolism

The functions of METTL3-mediated m^6^A methylation in insects are multifaceted, extending beyond reproductive growth and development to encompass physiological metabolism and behavioral performance. Particularly in social insects, METTL3 orchestrates collective metabolic states at the genomic level, thereby modulating caste-specific behaviors and phenotypic manifestations. The profound behavioral plasticity observed in social colonies is governed by a complex and precisely tuned regulatory network of gene expression.

A compelling paradigm is found in ants, where behavioral caste transitions are coupled with the fine-tuning of dopaminergic signaling within the brain. In ant cerebral cells, METTL3-mediated m^6^A modification targets the mRNAs encoding Dopamine receptor 1 (*Dop1*) and the Dopamine transporter (*DAT*), post-transcriptionally repressing their translation. This localized suppression of dopaminergic activity serves as a molecular switch, driving the behavioral shift from extranidal foraging (workers) to intranidal nursing (brood care by queens or specialized nurses) [38]. Such epitranscriptomic reprogramming is a cornerstone for the social organization and long-term sustainability of the colony.

In the migratory locust, *Locusta migratoria*, elevated cerebral expression of METTL3 empowers individuals to perceive and integrate population density cues, such as crowding or isolation, thereby facilitating rapid adaptive transitions. The LIM domain-containing transcription factor Lim3 (LOCMI14442) has been identified as a pivotal mediator in this m^6^A-regulated aggregation process. Specifically, *METTL3* promotes the targeted degradation of *Lim3* mRNA, which serves as a molecular trigger driving the transition from the solitary phase to the gregarious phase [6].

Social insects, exemplified by honeybees, exhibit remarkable phenotypic plasticity in caste differentiation. Comparison of m^6^A modification profiles in differentially expressed transcripts between queen and worker larvae at different developmental stages revealed a limited association between m^6^A and caste differentiation [39]. It has been documented that worker larvae possess significantly more hypermethylated m^6^A peaks than queen larvae, while caste-related transcripts display differential methylation [40]. Exposure to environmental stimuli can reshape transcriptional and epigenetic landscapes in worker tissues. These results collectively implicate RNA epigenetic regulation as an important mechanism underlying adult worker development, acting through tissue-specific methyltransferase expression that responds dynamically to age and behavioral demands [41]. In summary, METTL3 functions as an epitranscriptomic “molecular switch” that fine-tunes the expression of critical neurogenetic hubs within the insect brain, thereby shaping individual behavioral plasticity and social architecture. These findings illuminate the mechanistic foundations of epigenetic regulation in orchestrating collective behaviors and offer a transformative, epigenetics-centered strategy for pest management through the precision manipulation of pest behavioral trajectories.

Furthermore, METTL3-mediated m^6^A modification significantly enhances the adaptability of insects to complex environments by regulating biosynthetic metabolism and neural signaling. At the cellular level, the stress response is a core mechanism by which organisms counteract external adversities. During stress, intracellular transcription and protein synthesis processes are suppressed to alleviate cellular burden, thereby exhibiting enhanced antagonistic capacity. Research indicates that in a *Drosophila* heat shock (HS) model, METTL3 in the brain marks the mRNAs of heat shock chaperone proteins *Hsp70*, *DnaJ-1* (the mammalian Hsp40 homolog), and *stv* (the mammalian BAG3 homolog) with m^6^A modifications, thereby inhibiting their protein expression. This epitranscriptomic tagging effectively represses their protein expression, which paradoxically enhances overall stress tolerance and enables the flies to exhibit superior resilience to thermal extremes [42].

At the individual level, METTL3 is essential for the normal function of Mushroom Body (MB) neurons, a brain structure critical for cognition and memory in *Drosophila*. Deficiency in METTL3 leads to impaired short-term memory formation in the organism [43]. *Plutella xylostella*, an obligate pest of cruciferous plants, undergoes dynamic reprogramming of METTL3-mediated m^6^A methylation when switching between different host plants. This rapid epitranscriptomic regulation involves metabolic pathways such as steroid hormone synthesis, lipid metabolism, amino acid metabolism, and secondary metabolite production. Consequently, it enables the *P. xylostella* to balance resource allocation between stress adaptation and growth and reproduction, allowing for rapid adjustment to new host environments characterized by plant defense compounds and nutritional challenges [5].

At the organismal level, METTL3 exerts a profound influence on insect cognition and environmental resilience by modulating the neuro-transcriptomic landscape. The perception of and response to stimuli require the coordination and operation of the nervous system. METTL3 may confer greater environmental adaptability to insects by influencing the overall protein levels in neurometabolic pathways. This is supported by whole-transcriptome analyses, which show that METTL3 target genes are predominantly enriched in Gene Ontology (GO) terms such as “nervous system development” and “neurogenesis”, and that m^6^A-enriched neuronal transcripts are linked to key signaling pathways—including MAPK, Wnt, TGF-β, and Notch—that respond to various stress conditions [42]. In *P. xylostella* m^6^A RNA methylation regulates the trade-off between growth and defense by modulating juvenile hormone (JH) homeostasis. The m^6^A methyltransferases PxMETTL3 and PxMETTL14 suppress the expression of juvenile hormone esterase through m^6^A modification, resulting in elevated JH levels that alleviate fitness costs associated with resistance to *Bacillus thuringiensis*. This mechanism highlights the role of m^6^A in integrating post-transcriptional regulation with hormonal control in insect adaptive responses [43].

In conclusion, METTL3 constructs a robust, multi-dimensional regulatory network—spanning from cellular signaling to organismal behavior—that empowers insects to navigate fluctuating environments. By orchestrating host-adaptation strategies honed through co-evolutionary processes, METTL3 secures the fundamental biological pillars of growth, development, and reproduction. Ultimately, deciphering these m^6^A-dependent mechanisms provides critical insights into how insects successfully colonize, infest, and exploit their host plants.

### 5.3. Involvement in Insect Sex Determination

*N6*-methyladenosine modification serves as a cornerstone of epitranscriptomic regulation in insect sex determination. The core writer, METTL3, acts as a master coordinator of individual sexual dimorphism by modulating the alternative splicing (AS) of the primary sex-determining factor, Sex-lethal (*Sxl*). Genetic ablation of METTL3 results in profound molecular and phenotypic aberrations, most notably the defective splicing of *Sxl* pre-mRNA and associated neuro-behavioral abnormalities [44].

The *Sxl* protein is a female-specific, U-rich RNA-binding protein (RBP) whose functional expression is strictly governed by stage-specific splicing. While *Sxl* pre-mRNA is transcribed in both males and females, exon 3 is specifically retained only in males. This exon contains a premature termination codon; therefore, only female cells that achieve exon 3 skipping via alternative splicing can produce full-length, functional *Sxl* protein. Once functional full-length *Sxl* protein is produced, it initiates a positive feedback loop to maintain its own expression and that of its downstream target transformer (tra), thereby directing female differentiation. Concurrently, it suppresses the translation of male-specific lethal-2 (msl-2) mRNA to prevent dosage compensation mediated by upregulation of X-chromosome transcription, ultimately locking in the female developmental fate [45,46].

This high-fidelity sex-determination switch hinges on the tightly coordinated interplay between METTL3-mediated m^6^A modification and a suite of RNA-binding proteins (RBPs). In female cells, the RBP Nab2 serves as a key facilitator of exon skipping during the alternative splicing of *Sxl* pre-mRNA. To ensure this outcome, METTL3 catalyzes m^6^A deposition specifically onto exon 3 and its adjacent intronic flanking regions. The synergistic recruitment of Nab2 and the presence of these m^6^A marks act in concert to mechanically exclude exon 3 from the mature transcript. This dual-layered regulatory mechanism guarantees the fidelity of female-specific splicing, ultimately cementing the sexual identity of *Drosophila* [47,48].

Although direct reports on METTL3 in non-model insect sex determination remain limited, pivotal studies in *Drosophila* regarding its homolog, meiosis inducer 4 (*Ime4*), provide robust supportive evidence. Ime4 specifically catalyzes the m^6^A modification of *Sxl* pre-mRNA, a prerequisite for its female-specific alternative splicing and the subsequent execution of sexual differentiation [45]. From an evolutionary homology perspective, these findings underscore the deeply conserved role of the METTL3 methyltransferase family as an epitranscriptomic gatekeeper of sex determination. However, whether this conservation extends to high-level hierarchy in non-drosophilid insects remains an open question necessitating further empirical validation.

By orchestrating insect sex determination at the epitranscriptomic level, METTL3 and its homologs emerge as high-value targets for precision pest management. Manipulating the METTL3-mediated “sex-determination switch” offers a strategic avenue for distorting sex ratios within pest populations, thereby suppressing reproductive output. This provides a transformative theoretical framework for developing sustainable, “green” genetic control strategies—such as those based on gene drives or RNA interference—to mitigate pest-induced damage through epigenetic intervention.

### 5.4. Regulation of Neural Aging Resistance and Disease Defense Mechanisms in Insects

Emerging evidence underscores that METTL3 is vital for maintaining neurological integrity, thereby serving as a critical determinant of insect longevity. In aging *Drosophila* models, the depletion of METTL3 precipitates a marked decline in nervous system functionality, accompanied by the accumulation of the DNA damage marker ϒH2Av. This molecular instability ultimately leads to a significantly truncated lifespan, suggesting that METTL3 exerts a protective effect by shielding neurons from age-associated genomic insults [42]. The mechanistic underpinnings of this protective role involve the orchestration of DNA repair machinery. Studies in the rotifer *Brachionus plicatilis*—which provide comparative insights for insect aging—demonstrate that METTL3 upregulates key components of the non-homologous end joining pathway. By facilitating the repair of DNA double-strand breaks, METTL3 effectively mitigates genomic erosion and delays the senescence process. Furthermore, Furthermore, METTL3 is specifically required within *Drosophila* Mushroom Body neurons; its deficiency triggers accelerated, age-related deficits in short-term memory [49]. Collectively, these findings establish METTL3-mediated m^6^A modification as a central coordinator that links neuro-homeostasis with the systemic aging trajectory in insects.

Expanding beyond its neuroprotective functions, METTL3 plays a decisive role in orchestrating insect immune defenses and pathogen resistance. This antiviral capacity is vividly exemplified in the *Bombyx mori* response to BmNPV infection. Upon viral entry, the host’s heat shock protein 70 (Hsp70) is typically induced to facilitate viral replication. However, as a counter-regulatory mechanism, the silkworm rapidly activates METTL3, which catalyzes the m^6^A modification of *Hsp70* mRNA. This modification impairs translational efficiency, thereby starving the virus of the structural protein VP39 and effectively aborting BmNPV proliferation [7].

In addition to antiviral immunity, METTL3 is a central player in the evolutionary arms race against bacterial pathogens. During its long-term co-evolution with *Bacillus thuringiensis*, the diamondback moth, *P. xylostella*, has developed highly resistant strains characterized by the constitutive upregulation of *PxMETTL3*. Elevated METTL3 levels enhance the m^6^A tagging of *PxJHE* (Juvenile Hormone Esterase) mRNA, repressing its expression and resulting in systemic JH hyper-accumulation. This elevated JH then synergizes with 20E to trigger the MAPK signaling cascade, which modulates the phosphorylation of the transcription factor FTZ-F1. This regulatory axis orchestrates the differential expression of diverse Bt-toxin receptors, thereby conferring robust resistance to the Cry1Ac toxin [4].

Notably, METTL3-mediated m^6^A modification extends its regulatory reach beyond direct host immunity to the delicate maintenance of immune homeostasis within viral vectors. Following infection of *Laodelphax striatellus*, an insect vector, by the rice black-streaked dwarf virus (RBSDV), the virus replicates its mRNA, which is translated into viral proteins. These components assemble into new virions in the midgut epithelial cells of *L. striatellus*. Infected *L. striatellus*, often present in high population densities, then spread the virus to rice crops during their activity. Stable m^6^A modification across the virus, vector, and host helps maintain a viral threshold within the insect vector necessary for persistent transmission, allowing the insect to tolerate a certain viral load without apparent adverse effects. After RBSDV infection, METTL3 expression is activated in *L. striatellus* midgut epithelial cells, leading to an overall increase in m^6^Alevels. This elevated m^6^A helps maintain RBSDV at a lower titer, suggesting that the insect vector employs METTL3-mediated m^6^A modification of viral replication-related mRNAs to prevent excessive viral accumulation, thereby protecting itself from harm [50].

Furthermore, as the METTL3 homolog in *Drosophila*, DmIme4 potentially regulates the alternative splicing of the mRNA transcript encoding the filamentous actin-binding protein Chic. This regulation, whether direct or indirect, contributes to the formation of the cyst cell structure that provides a physical barrier during spermatogenesis [32].

Collectively, these findings underscore METTL3’s status as a master regulatory node that empowers insects to navigate multifaceted internal and external challenges. It offers a sophisticated framework for understanding how m^6^A modification governs immune surveillance and structural barrier integrity.

Although METTL3 expression can stimulate immune responses in insects, its immunoregulatory function exhibits cell type-specificity. For instance, in the *Drosophila* brain, upregulation of *METTL3* is generally beneficial to neurons but detrimental to glial cells. Therefore, under specific conditions, knocking down *METTL3* in glial cells can also produce disease-resistant effects in the organism. In glial cells of the *Drosophila* brain, aging and disease progression are accompanied by abnormal phosphorylation and accumulation of the microtubule-associated protein tau, leading to pathological tau enrichment in cells. This results in shortened lifespan and brain tissue vacuolization in the organism. Downregulation of *METTL3* in glial cells increases the phosphorylation level of GSK3β at Ser9, significantly reducing pathological tau foci and thereby extending the lifespan of the glial tauopathy model [42].

**Figure 9:**
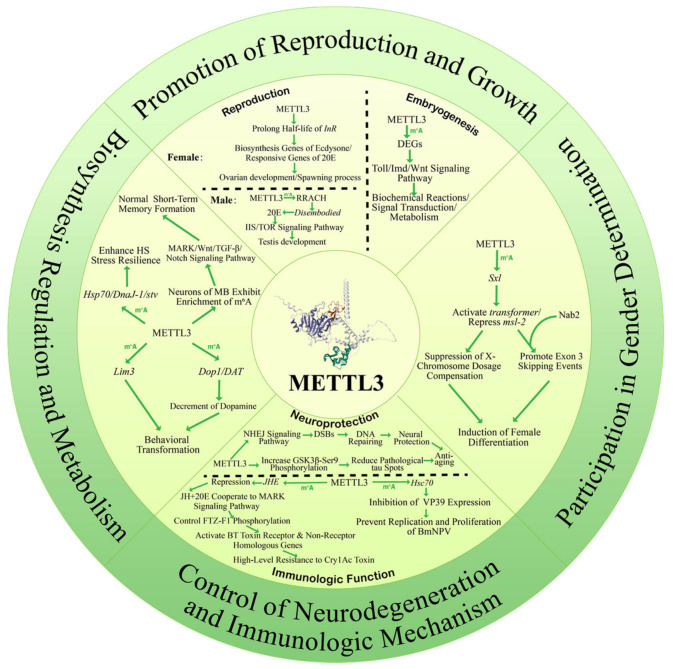
Study on the role of METTL3 in insects. METTL3 coordinates diverse physiological processes through m^6^A modification of distinct target genes. In reproduction and development, METTL3 promotes gonadal maturation by upregulating 20-hydroxyecdysone (20E) expression, and concurrently activates Toll/Imd/Wnt signaling pathways to orchestrate growth, immune surveillance, and metabolic homeostasis. In the process of biological responses, METTL3 mediates behavioral plasticity and stress resilience via methylation of *Lim3*, *Dop1*, *DAT*, *Hsp70*, *DnaJ-1*, and *stv*, and facilitates short-term memory formation through m^6^A enrichment in the mushroom body (MB) that regulates MARK/Wnt/TGF-β signaling. In sex determination, METTL3 participates in the methylation-mediated regulation of *Sxl* and *msl-2*, thereby influencing sexual differentiation. In neurobiology, METTL3 enhances anti-aging capacity by activating the NHEJ DNA repair pathway and increasing GSK3β-Ser9 phosphorylation. Lastly, METTL3 modulates immune and detoxification responses through methylation of JHE (conferring bacterial tolerance) and Hsc70 (suppressing viral replication), highlighting its central role as a pleiotropic regulator integrating developmental, behavioral, and stress-adaptive programs across insect taxa.

In summary, METTL3 orchestrates a comprehensive defense architecture that safeguards insect immunity and physiological homeostasis through a tripartite regulatory strategy: suppressing viral pathogenesis, modulating bacterial toxin resistance, and maintaining immune tolerance in viral vectors, alongside the reinforcement of structural physical barriers. While the granular molecular trajectories of METTL3-mediated regulation continue to be mapped, these pioneering insights undoubtedly pave the way for a dual-purpose biotechnological frontier. This includes the development of “epitranscriptome-targeted” green pest management strategies for deleterious species and the implementation of precision health-monitoring frameworks for beneficial insects, such as pollinators and silk-producers (Figure 9).

## 6. Conclusions and Perspectives

This study leveraged systematic bibliometric methodologies, employing CiteSpace to analyze a decade of METTL3-related scholarship retrieved from the Web of Science Core Collection. Through high-resolution mapping of co-authorship networks, keyword co-occurrence, and structural centrality, we delineated a research landscape organized around three primary clusters: first, the role of METTL3-mediated m^6^A modification in metabolic homeostasis, encompassing protein phosphorylation, lipid biosynthesis, and neuromodulation; second, its pivotal involvement in host–pathogen dynamics during viral and bacterial immune challenges; and third, the integration of high-throughput genomic technologies to decode the molecular architecture and predictive targets of METTL3, a trend that continues to expand the field’s horizons.

Previous bibliometric investigations have systematically explored research trends in m^6^A modification within mammalian systems, cancer biology, and inflammatory diseases, providing valuable quantitative frameworks for understanding knowledge evolution and collaborative networks in those fields [9,51,52].. However, a bibliometric synthesis specifically focused on insect m^6^A regulation—and particularly on the core methyltransferase METTL3—has been lacking. To address this gap, the present study provides the first comprehensive bibliometric mapping of insect epitranscriptomics, integrating both broad m^6^A-related research and METTL3-centered mechanistic studies. By comparing our findings with prior bibliometric analyses in other biological contexts, we identify both conserved patterns of research expansion and insect-specific features, such as the prominence of developmental and behavioral phenotypes as major thematic clusters. This comparative perspective not only validates the robustness of our analytical approach but also highlights the unique position of insect METTL3 research as an emerging frontier that bridges fundamental epitranscriptomic mechanisms with organismal adaptation and applied entomology.

While providing a comprehensive overview, the inherent limitations of this study must be acknowledged. Our data synthesis was constrained by the coverage of the primary database and specific search algorithms, relying solely on the Web of Science Core Collection. Furthermore, the intrinsic algorithmic parameters of CiteSpace in clustering granularity, burst detection, and temporal visualization may introduce specific biases in trend representation. Nevertheless, this study successfully charts the evolutionary trajectory of METTL3—from its initial identification in fundamental biochemical pathways to its emergence as a master regulator of the organismal life cycle. These findings offer a robust theoretical framework and methodological roadmap for navigating the future of epitranscriptomic research.

Building upon the aforementioned analytical results, this study focuses specifically on research progress regarding METTL3-mediated m^6^A in insects. Recent scientific investigations have clearly established that METTL3-mediated m^6^A methylation is a crucial epitranscriptomic mechanism regulating multiple core physiological processes in insects. By constructing multi-tiered regulatory networks, it participates in the precise control of gene expression, encompassing biological processes such as reproductive development, neural function, metabolic homeostasis, immune defense, behavioral plasticity, and environmental adaptation. Of particular note is that the biological effects of METTL3 in insects are not simply linear relationships between gene expression and phenotype; rather, they exhibit highly context-dependent multidimensional regulatory characteristics. This complexity is reflected in METTL3’s ability to regulate the developmental fate of different cell types within the same organism, drive phenotypic variation among individuals of the same species, and even govern the emergence of collective behaviors in social insect colonies. In summary, METTL3 achieves fine-tuned and precise epitranscriptomic regulation of insects across multiple dimensions and aspects, establishing itself as a significant focus in insect research.

The high degree of cross-species conservation observed in METTL3 suggests that novel regulatory paradigms uncovered in insect models may provide profound insights into its physiological and pathological roles in mammals. In the realm of oncology, METTL3 has already been established as a functional “double-edged sword” in malignancies such as colorectal cancer and acute myeloid leukemia. By modulating cell proliferation, apoptosis, and the epithelial–mesenchymal transition, *METTL3* dictates tumor progression and chemoresistance, underscoring its immense potential as a precision therapeutic target.

In summary, the integration of functional evidence with the evolutionary trajectories mapped by our bibliometric analysis solidifies METTL3 as an indispensable cornerstone of epigenetic research. Our keyword co-occurrence and cluster mapping visually underscore the systematic nature of METTL3’s reach, while timeline visualizations effectively trace the field’s maturation from deciphering fundamental catalytic mechanisms to exploring expansive physiological networks.

Moving forward, future investigations must bridge the gap between macro-level bibliometric trends and micro-level mechanistic data. The goal is not only to decode insect-specific regulatory motifs but also to elucidate the universal regulatory patterns conserved throughout evolution. Such an endeavor is poised to establish a “reverse translational medicine” paradigm, where discoveries in insect models catalyze breakthroughs in mammalian medical research. This approach provides a visionary theoretical foundation for translating fundamental epitranscriptomic discoveries into transformative strategies for human disease prevention and clinical intervention.

## Data Availability

Data sharing not applicable to this article as no datasets were generated or analyzed during the current study.

## Abbreviations

20E	20-Hydroxyecdysone
AS	Alternative Splicing
BmNPV	*Bombyx mori* Nucleopolyhedrovirus
CDS	Coding Sequence
DEGs	Differentially Expressed Genes
GO	Gene Ontology
Hsp70	Heat Shock Protein 70
JH	Juvenile Hormone
m^6^A	*N*^6^-Methyladenosine
MAPK	Mitogen-Activated Protein Kinase
METTL3	Methyltransferase-like 3
METTL14	Methyltransferase-like 14
PRISMA	Preferred Reporting Items for Systematic Reviews and Meta-Analyses
RBP	RNA-Binding Protein
SAM	S-Adenosylmethionine
Sxl	Sex-lethal
